# Additional Insights into the REPLICA-PH Study

**DOI:** 10.21470/1678-9741-2021-0591

**Published:** 2023-06-14

**Authors:** Ashok Kumar Karamsi

**Affiliations:** Department of Cardiac Anaesthesia, Sri Padmavathi Children’s Heart Center, Tirupati, India. E-mail: ashok.medickaramsi@gmail.com

Dear Editor,

The recently published “Retrospective Evaluation of Platelet-Leukocyte Indices and Cardiac Surgical Outcomes in Acyanotic Heart Disease Patients with Pulmonary Hypertension (REPLICA-PH)” study by Walian et al.^[1]^ on the prognostic role of platelet-leukocyte indices in congenital cardiac surgical subset with pulmonary hypertension (PH) is indeed interesting, and the authors should be congratulated for their admirable endeavors. However, I wish to bring forth few additional insights into the topic that could be beneficial to the readers of the BJCVS.

Inflammatory prognostication using hematological cell lines is an evolving field with the novel composite indices being increasingly studied across diverse clinical scenarios^[2,3]^. While Walian et al.^[1]^ have evaluated neutrophil-lymphocyte ratio (NLR), platelet-lymphocyte ratio, and systemic immune-inflammation index (SII = neutrophil × platelet/lymphocyte) in their retrospective analysis, Fois et al.^[2]^ recently described other hematological inflammatory indices such as the systemic inflammation response index (SIRI = NLR × monocyte) and the aggregate index of systemic inflammation (AISI = NLR × platelet × monocyte or SII × monocyte) in the setting of coronavirus disease 2019 (or COVID-19).

Recent research outlines monocyte corpuscular lineage as an important contributor to an ongoing systemic inflammatory process^[4]^. Hence, a comparative account of SIRI and AISI in the favorable and poor-outcome groups in the Walian et al.^[1]^ analysis could have added an incremental value.

Moreover, the lack of data on pulmonary vascular resistance (PVR) in the index of the retrospective study also deserves equal attention. The research group circumspectly labels the study cohort to be suffering from preoperative PH and not pulmonary artery hypertension, refraining to *“err on either side of the pulmonary capillary”*. The provision of the PVR data could have substantiated the authors’ proposition of inflammatory links of proliferative remodeling of pulmonary vasculature in the congenital cardiac surgical subset with an underlying increased pulmonary blood flow^[5]^.

Although the authors excluded multisystem syndromic association from their analysis^[1]^, future research endeavors exploring haematological inflammatory prognostication in a dedicated syndromic subset can yield potentially interesting results.

## References

[1] Walian A, Kohli JK, Magoon R, Kashav RC, Shri I, Dey S (2022). Retrospective evaluation of platelet-leukocyte indices and
cardiac surgical outcomes in acyanotic heart disease patients with pulmonary
hypertension (REPLICA-PH). Braz J Cardiovasc Surg.

[2] Fois AG, Paliogiannis P, Scano V, Cau S, Babudieri S, Perra R (2020). The systemic inflammation index on admission predicts in-hospital
mortality in COVID-19 patients. Molecules.

[3] Magoon R, Jain A (2021). Haematological inflammatory prognostication in COVID-19: points
to ponder!. Am J Emerg Med.

[4] Kapellos TS, Bonaguro L, Gemünd I, Reusch N, Saglam A, Hinkley ER (2019). Human monocyte subsets and phenotypes in major chronic
inflammatory diseases. Front Immunol.

[5] Chemla D, Lau EM, Papelier Y, Attal P, Hervé P (2015). Pulmonary vascular resistance and compliance relationship in
pulmonary hypertension. Eur Respir J.

